# Data supporting the hierarchically activated deformation mechanisms to form ultra-fine grain microstructure in carbon containing FeMnCoCr twinning induced plasticity high entropy alloy

**DOI:** 10.1016/j.dib.2022.108052

**Published:** 2022-03-12

**Authors:** Mohsen Saboktakin Rizi, Hossein Minouei, Byung Ju Lee, Hesam Pouraliakbar, Mohammad Reza Toroghinejad, Sun Ig Hong

**Affiliations:** a Energy Functional Materials Laboratory (EFML), Department of Materials Science and Engineering, Chungnam National University, Daejeon, Republic of Korea; b Department of Materials Engineering, Isfahan University of Technology, Isfahan 84156-83111, Iran

**Keywords:** Ultrafine-grained, Hierarchical structure, Twinning induced plasticity, Microband induced plasticity, Shear banding, High entropy alloy

## Abstract

This article presents data regarding the research paper entitled “Hierarchically activated deformation mechanisms to form ultra-fine grain microstructure in carbon containing FeMnCoCr twinning induced plasticity high entropy alloy [1]”. In this article we provide supporting data for describing the associated mechanisms in microstructure evolution and grain refinement of a carbon-doped TWIP high-entropy alloy (HEA) during thermomechanical processing. Microstructural characterization before and after deformation was performed using scanning electron microscope (SEM) outfitted with EBSD detector and transmission electron microscopy (TEM) were used for microstructure observation and investigation of nanostructure evolution during deformation. Inverse pole figure (IPF) map, grain boundary map and kernel average misorientation map (KAM) were used for systematic analysis of nanostructural evolution and deformed heterostructure consisting of hierarchical mechanical twinning, shear-banding, microbanding and formation of strain-induced boundaries (SIBs).

## Specifications Table

**Table:** 

Subject	Metals and alloys
Specific subject area	Nanostructural evolution and deformation of high entropy alloys (HEAs)
Type of data	Table (mechanical properties, EDX profiles), Chart (Misorientation angle), Figure (EBSD, TEM and STEM)
How data were acquired	- Mechanical properties data by tensile testing at room temperature - Microstructure characterization by scanning electron microscope (SEM) and - Nano structure characterization by transmission electron microscope (TEM). - The compositional was investigated using energy dispersive spectroscopy (EDS) in scanning transmission electron microscopy (STEM) mode.
Data format	Raw data: SEM, TEM, STEM, EBSD images, Stress-strain curves.
Parameters for data collection	- Mechanical responses of the as-received and as-rolled samples were examined via United SFM-10.5-ton tensile testing machine at room temperature and strain rate of $1\;\times 10^{-3}s^{-1}$. - Microstructures were investigated by electron back scattered diffraction (EBSD) system (Oxford Instruments, UK) attached to a FE-SEM (Helios, Pegasus, FEI). BSD was performed using step size of 70 nm at an accelerating voltage of 20 kV. EBSD data were analysed using EDAX/TSL OIM data collection software. - Nanostructures were analyzed by TEM with a FEI Tecnai G2 F30 S-TWIN operated at an acceleration voltage of 200 kV.
Description of data collection	EBSD samples were cut, ground down to a 2000-grit SiC paper and electro-polished at room temperature. specific TEM foils were carried out using twin jet polishing machine in the solution of 10% perchloric acid and 90% methanol at −30 °C under the voltage of 24 V.
Data source location	Institution: Chungnam National University City/Town/Region: Daejon Country: Republic of Korea
Data accessibility	Data are with the article. The raw data are in the Mendeley Data repository. https://doi.org/10.17632/m6z98wy24x.1
Related research article	M. S. Rizi, H. Minouei, B. J. Lee, H. Pouraliakbar, M. R. Toroghinejad, and S. I. Hong, Hierarchically activated deformation mechanisms to form ultra-fine grain microstructure in carbon containing FeMnCoCr twinning induced plasticity high entropy alloy, Mater. Sci. Eng. A. 824 (2021) 141803. https://doi.org/10.1016/j.msea.2021.141803.

## Value of the Data

• Data on deformation mechanisms of carbon-doped FeMnCoCr high entropy alloys (HEA) are useful for researchers in metals and alloys research community particularly in the field of mechanical performance of medium and high entropy alloys.
• The present data provides insight into the alloy design strategy to overcoming strength-ductility trade-off in FCC high entropy alloys by deriving bimodal grain size through thermomechanical processing.
• The conjunction results of ultimate tensile strength (UTS) and ductility of the present alloy with various high/medium entropy alloys and steels would provide a useful information on the correlation of the gradient microstructure and mechanical performance of high entropy alloys and steels.

## 1 Data Description

**Fig. 1:**
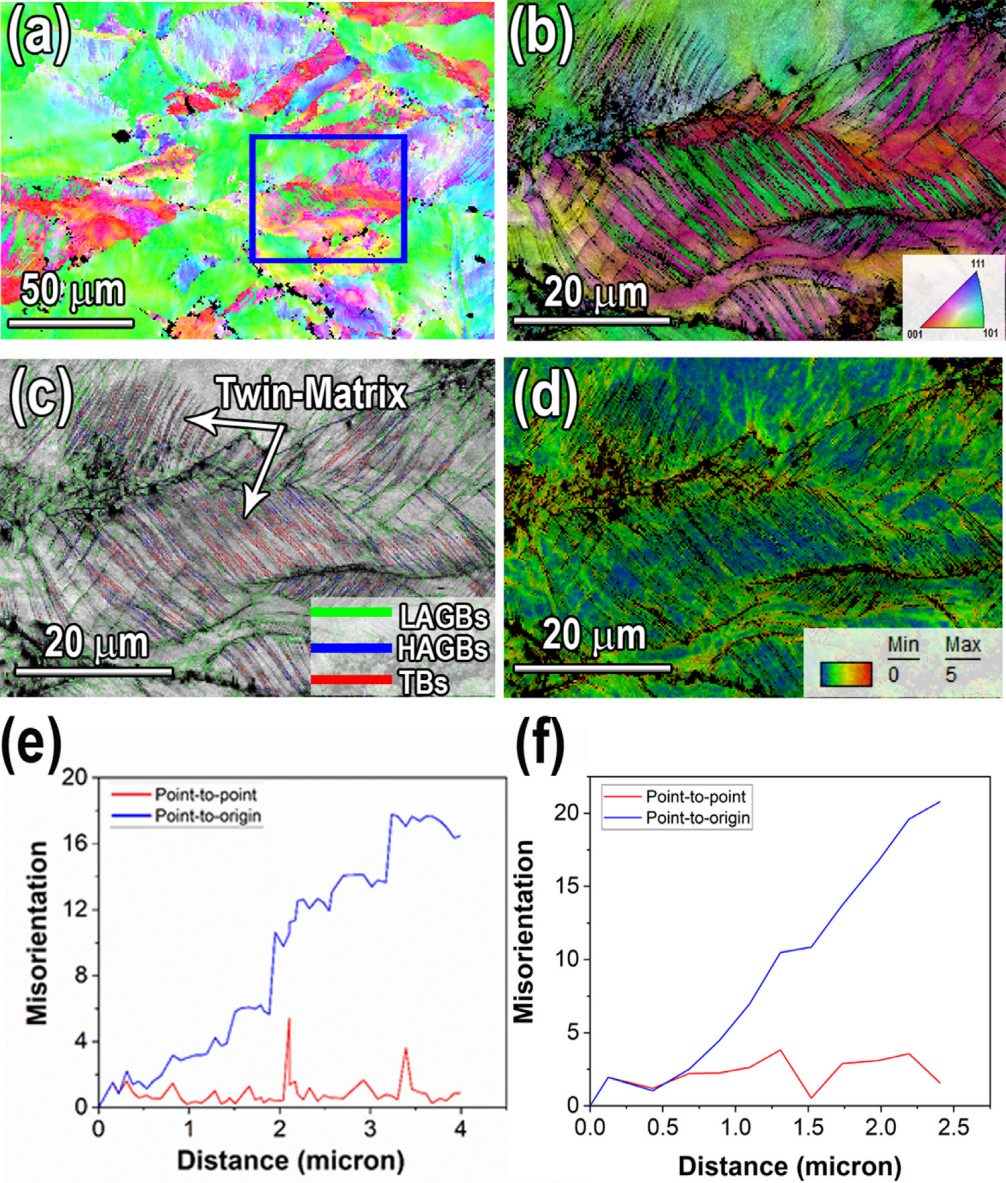
(a) EBSD inverse pole figure (IPF) map of the 32% cold rolled Fe_39.5_Mn_40_Co_10_Cr_10_C_0.5_ HEA (b) IPF map of the area enclosed to blue rectangle of Fig. 1(a). (c) IQ map of low angle boundaries (2°-15°), high angle boundaries (15°-180°) and Σ3 twin boundaries. (e) the misorientation angle measurement along slip bands. (f) The corresponding misorientation angle profile along to A-B which corresponds to shear banding formed in grain C in fig 1 of the research article [1].

Microstructure details and corresponding EBSD maps of the carbon containing Fe_39.5_Mn_40_Co_10_Cr_10_ HEA subjected to 32% cold roll reduction is shown in Fig. 1. The EBSD maps and corresponding misorientation angle profiles were presented in the Mendeley Data repository (“Fig. 1-EBSD grain boundaries map.tif,” Fig. 1-EBSD IPF map.tif, Fig. 1-EBSD KAM map.tif and “Fig. 1-Misorientation angle profiles. .xlsx”).

Cold deformed microstructure of Fe_39.5_Mn_40_Co_10_Cr_10_C_0.5_ HEA in Fig. 1 (a)-(c) contains some microstructural heterogeneities such as slip bands, deformation twinning and shear banding which associated with large strain gradients. In order to differentiate local misorientations and orientation gradients in the regions of heterogeneities, EBSD combined with a kernel average misorientation (KAM) map is used. In this study misorientation angle was also used for quantitative measurement of plastic strain at heavy deformed microstructure. As seen in Fig. 1(d) low KAM values was identified in the matrix which dominated by blue. In contrast, as shown by green in Fig. 1d high KAM values appeared in the regions of slip bands and twin boundaries (TBs) which related to the high local strain in the regions enclosed by the slip bands and TBs. Based on definition of misorientation, $\theta <15^{\circ }\;$is considered as a low angle grain boundaries (LAGB) and $\theta >15^{\circ }\;$is considered as a high angle grain boundaries (HAGB) [2,3]. The point-to-origin and point-to-point misorientation angle profiles of deformation bands in Fig. 1(e) exhibit a misorientation angle around 2-18°. On the other hand, deformation twins in large grains exhibited a misorientation angle of 60° with respect to the fcc matrix [3] Fig. 1. (f) shows the increase of the misorientation angle along A-B line close to the nano-shear bands (in Fig 3(e1) -(e2) of the research article [1]) which also implied high dislocation density near the shear bands [1].

**Fig. 2:**
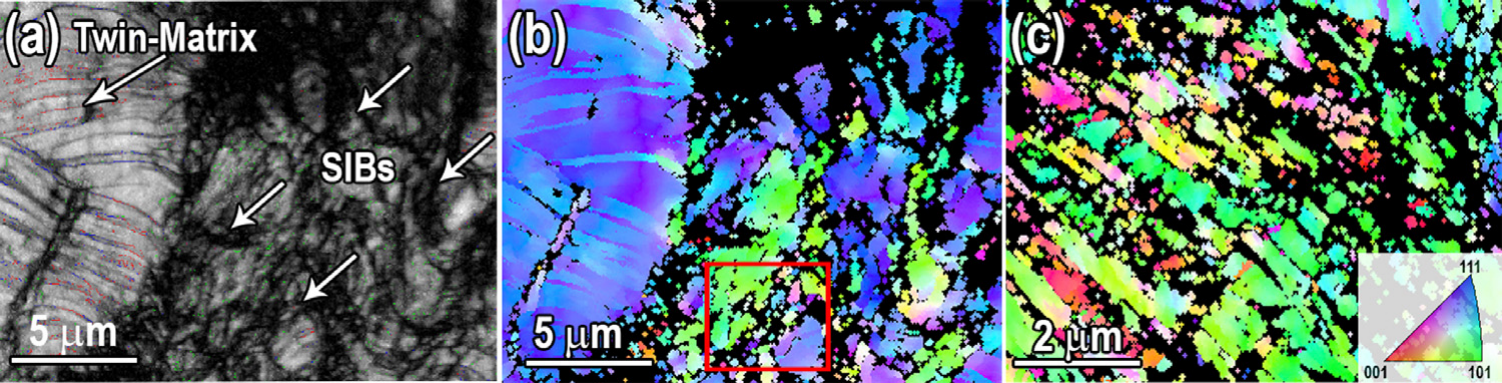
(a) and (b) IQ map and EBSD IPF of the development of the deformation-induced grain boundaries (c) continuous dynamic recrystallization (CDRX) at deformation-induced boundaries in the red rectangle in (b) for sample subjected to 84% cold rolling reduction.

**Fig. 3:**
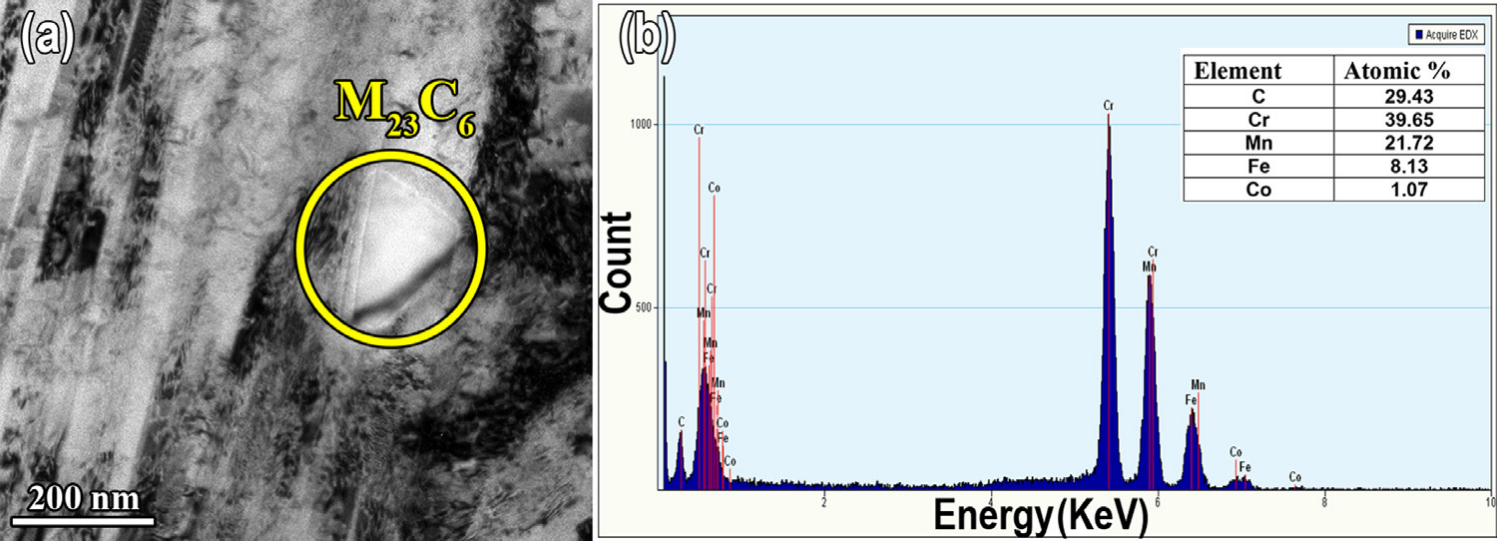
(a) TEM bright-filed images of strain-induced precipitates in carbon-containing Fe_39.5_Mn_40_Co_10_Cr_10_ HEA after cold rolling (b) EDS analysis of the M_23_C_6_ precipitates.

Fig. 2 displays the EBSD images of the 84% cold rolled sample. The EBSD maps were presented in the Mendeley Data repository (“Fig. 2-EBSD IQ map of 84% cold rolled reduction.jpg”, “Fig. 2-EBSD IPF map of 84% cold rolled reductio.jpg” and “Fig. 2-EBSD IPF of dynamic recrystallization.jpg”). As can be seen in Fig. 2, deformation-induced boundaries developed in heavily deformed microstructure (Fig. 2, 2(b)). The elongated grain is subdivided into different domains and fine grain structure formed by continuous dynamic recrystallization (DRX) within the deformation-induced boundaries (Fig. 2 (c)) [1]. The bright-field TEM image and EDS analysis of the precipitations were presented in the Mendeley Data repository (“Fig. 3-TEM.jpg” and “Fig. 3- TEM EDS analysis of carbides.txt”). TEM-EDS analysis of the strain-induced precipitation during the cold roll deformation is shown in Fig. 3(a), 3(b). The EDS results showed that these precipitates are Cr, Mn and carbon rich.

**Fig. 4:**
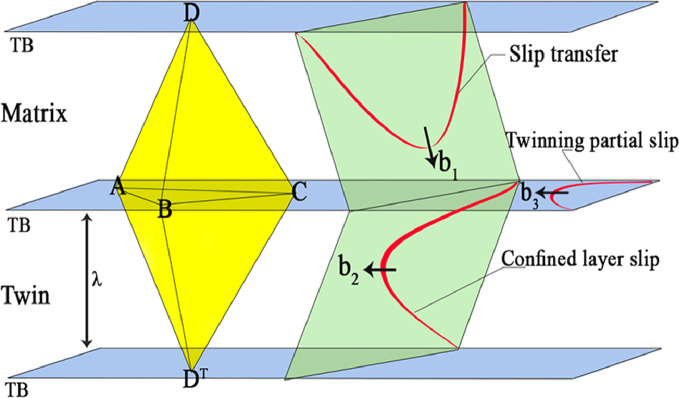
Different types of dislocation-twin boundary interactions, mode I: burgers vector and slip plane make angles with TB, mode II: burgers vector is parallel to TB but slip plane makes an angle with TB, mode III: burgers vector and slip plane are along to twin boundary [4].

Fig. 4 presents the double Thompson tetrahedron and different types of dislocation-twin boundaries interactions in face centred cubic alloys. Dislocation–TB interactions will be largely affected by the twin thickness and dislocation sources. Most models of dislocation-TB interactions are based on the loading conditions and various interaction modes involving twinning partial dislocations, slip transfer and confined-layer slip have been interpreted for TBs-dislocation interactions.

**Fig. 5:**
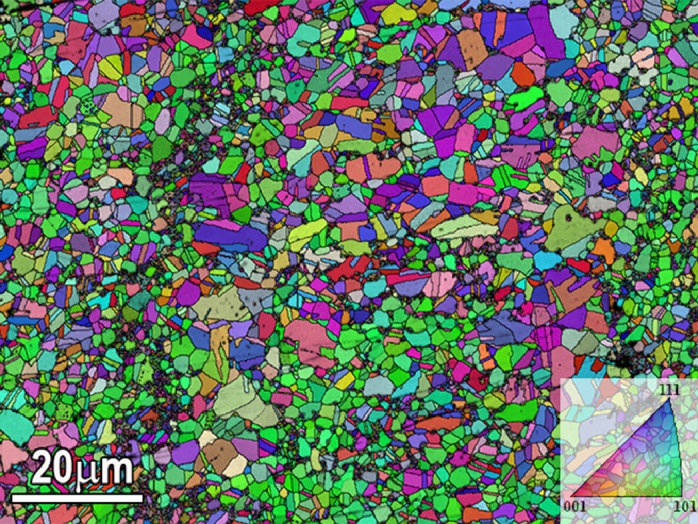
EBSD IPF map of specimen annealed at 850 °C after 84% rolling reduction, showing heterogeneous bimodal microstructure.

**Fig. 6:**
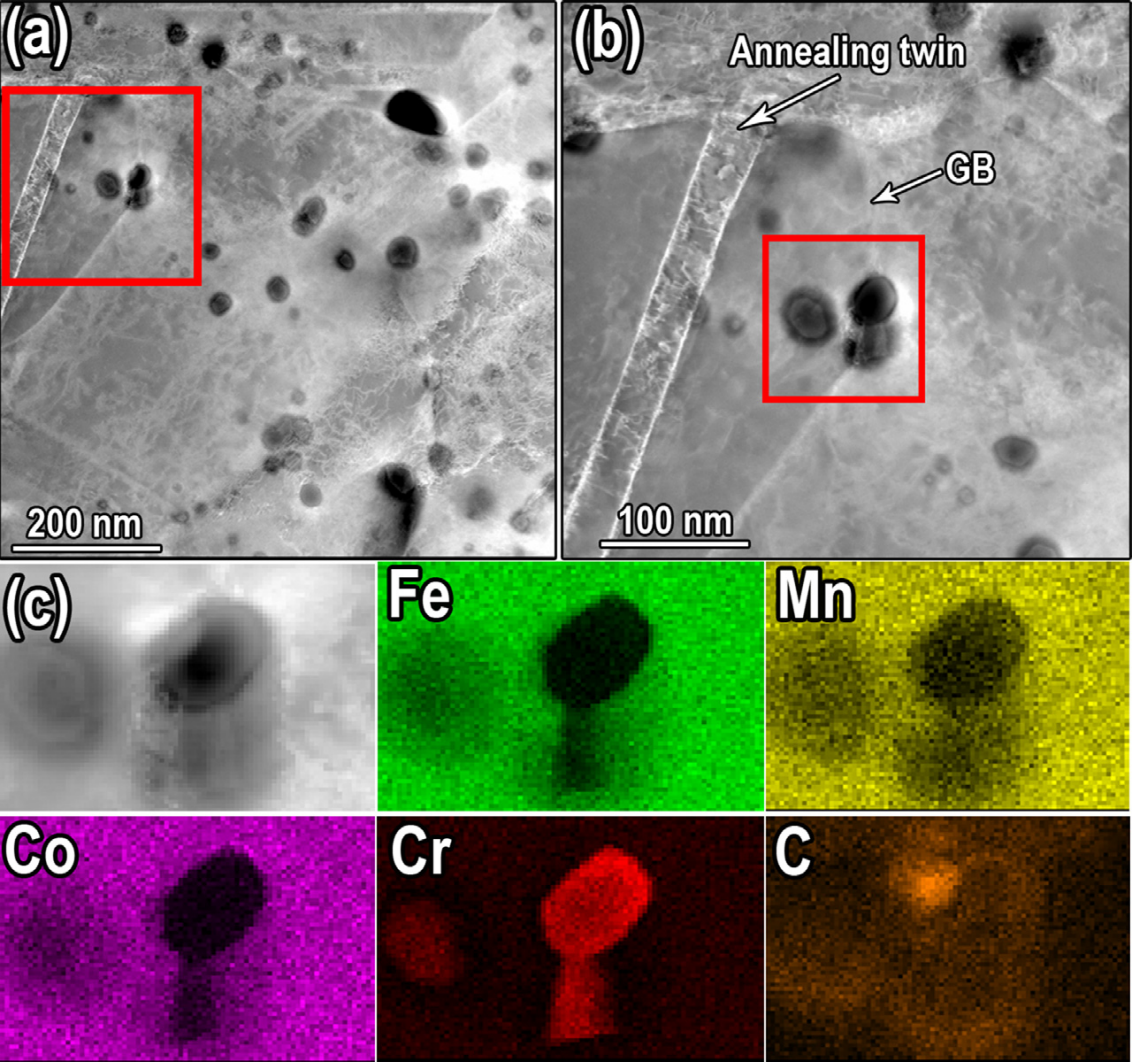
STEM observation of M_23_C_6_ distribution for specimen annealed at 850 °C for 30 min after 84% rolling reduction. (b) Enlarged STEM image of the region enclosed by a red rectangle in (a) exhibits precipitation at annealing twin boundaries and grain boundaries. (c) EDS elemental mapping images of Fe, Mn, Co, Cr and carbon for precipitations at rectangular region in (b).

The microstructure of the annealed HEA with the pre-rolling reductions of 84% is shown in Fig 5 and was presented in the Mendeley Data repository (“Fig. 5-EBSD IPF map of bimodal structure.jpg”). The EBSD IPF map of the annealed sample illustrate the development of heterogeneous bimodal structure consists of ultra-fine grains (with grain size of .5 µm) and larger grains (with grain size of 3µm). Fig. 6 exhibits the STEM nanostructure (a and b) and EDS mapping images of Fe, Mn, Co, Cr and carbon (Fig. 6(c)) of the annealed specimen at 850 °C for 30 min after 84% pre-rolled. Furthermore, STEM images were presented in the Mendeley Data repository (“Fig. 6-STEM observation of M_23_C_6_ distribution” and “Fig. 6-Enlarged STEM image of M_23_C_6_ distribution.jpg”). In the STEM image (a) and (b), nano-scale precipitation (average size of 70 nm) at grain boundaries and twin boundaries are shown. EDS analysis in Fig. 4(c) shows that precipitations are enrich of Cr, Mn and carbon.

**Fig. 7:**
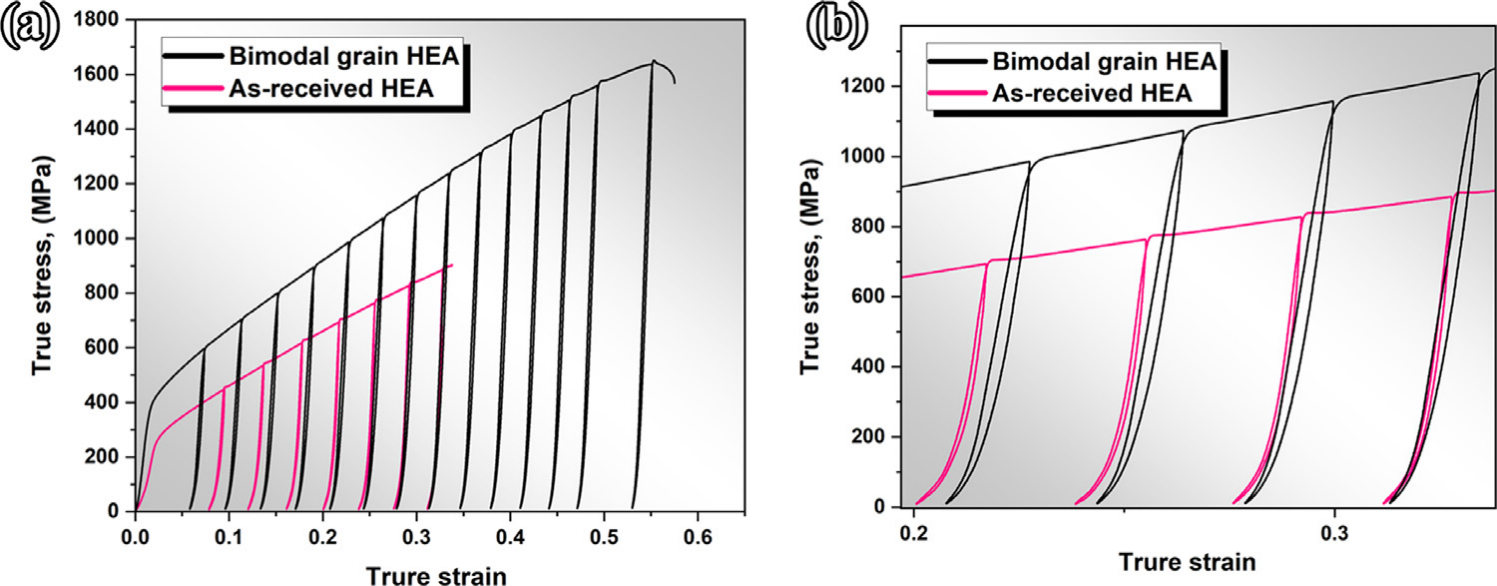
(a) The Load-unload-reload true stress-strain behaviour of the as-received and bimodal HEAs at the strain rate of $1\;\times 10^{-3}s^{-1}$(b) The Enlarged hysteresis loops of the as-received and bimodal HEAs at true strain of 0.2-0.35.

To manifest the effect of grain size (larger grains and ultra-fine grains) on deformation mechanisms of sample with bimodal structure, Loading-unloading-reloading (LUR) tensile tests were conducted and was presented in the Mendeley Data repository (“Fig. 7-load-unload-reload true stress-strain curves. xlsx”) Fig. 7(a, b) presents the LUR test curves for as-received (as homogenized) sample and thermomechanlcally processed HEA with bimodal structure. As shown in Fig. 7(a) The Fe_39.5_Mn_40_Co_10_Cr_10_C_0.5_ HEA with heterostructure exhibits superior strength and ductility, which is mainly attributed to the hetero-deformation induced (HDI) strengthening. Moreover, the hysteresis loops of the alloy with bimodal grain size in Fig. 7(b) is much wider than that of homogenized sample with homogeneous large grains which is associated with the Bauschinger effect.

Data of mechanical properties of Fe_39.5_Mn_40_Co_10_Cr_10_C_0.5_ HEA with heterogenous bimodal structure and some recently investigated TWIP-TRIP high entropy alloys and steels are summarized in Table 1. It was shown that the bimodal heterogeneous structure formed by thermomechanical processing contributes to strength-ductility enhancement in carbon containing Fe_39.5_Mn_40_Co_10_Cr_10_ HEA.

**Table 1:** Data on mechanical properties of the C-doped Fe_39.5_Mn_40_Co_10_Cr_10_ high entropy alloy and other TWIP/TRIP alloys.

Alloys (Grain size)	UTS (MPa)	Elongation (%)	Ref
Fe_39.5_Mn_40_Co_10_Cr_10_C_0.5_ (bimodal structure 0.5-3 µm)	840	88	This work
Fe_40_Mn_40_Co_10_Cr_10_ (130 µm)	544	42	[5]
Fe_40_Mn_40_Co_10_Cr_10_ (108 µm)	500	58	[6]
(Fe_40_Mn_40_Co_10_Cr_10_)_96.7_C_3.3_ (95 µm)	600	60	[7]
(Fe_40_Mn_40_Co_10_Cr_10_)_96.7_C_3.3_ (60 µm)	935	74.4	[7]
Fe_40_Mn_27_Ni_26_Co_5_Cr_2_ (12 µm)	645	50	[8]
Fe_50_Mn_25_Cr_15_Co_10_ N_1.6_ (12.7µm)	1050	80	[9]
FeMn_30_Co_10_Cr_10_(4.7µm) TWIP-TRIP	870	75	[10]
FeMn_30_Co_10_Cr_10_C_0.5_(4 µm) TWIP-TRIP	870	75	[11]
FeMn_30_Co_10_Cr_10_C_0.5_ (Nanostructure)TWIP-TRIP	1000	35	[12]
Fe_45_Co_30_Cr_10_V_10_Mn_5_(9.8 µm) TRIP	802	66	[13]
Co_35_Cr_25_Mn_15_Ni_15_Fe_10_ (11.2 µm) TRIP	806	76	[14]
Ni based alloy (50 nm)	684	44	[15]
FeMnCoCrNi (7.9 µm)	491	66	[16]
CoCr_0.25_FeMnNi (150 µm)	795	58	[17]
FeMnCoCrNiC_0.5_ (4.7 µm)	569	48	[16]
CrCoNi (16 µm)	750	30	[18]
Fe_36_Mn_36_Ni_9_Cr_9_Al_10_C_1.5_ (26 µm)	755	49	[19]
Al_0.3_Cu_0.5_CrFeNi_2_C_0.07_ (100 µm)	904	39	[20]

## 2 Experimental Design, Materials and Methods

A non-equiatomic Fe_39.5_Mn_40_Co_10_Cr_10_C_0.5_ (at%) HEA was cast using vacuum induction melting of Fe, Mn, Co, Cr elements. Purity of used element was higher than 99.9% and carbon black used as a source of 0.5 at% C [1]. The 10 Kg of as cast ingot was remelt for 5 times to ensure the compositional homogeneity. For break down the cast structure and further homogenization, the as-cast ingot was hot-rolled at 900 °C to a thickness reduction of 60%. In order to induce grain refinement after homogenization of hot rolled samples at 1200 °C for 2 hours in Ar atmosphere the alloy was cold-rolled to thickness reduction of 32–84%. Post-cold deformation annealing at 850 °C for 30 min was conducted for 84% cold rolled samples followed and water-quenched. Uniaxial tensile tests were performed using United SFM-10.5-ton tensile testing machine at room temperature and strain rate of $1\;\times 10^{-3}s^{-1}$ on the as- received and as-rolled samples [1]. Dog-bone shaped tensile specimens with a gauge length of 9 mm and a width of 3.4 mm were used. Tensile tests were executed aligned into rolling direction. Microstructures and nanostructures were examined by EBSD and TEM. The TEM samples were mechanically ground to a thickness of 70 µm using 120-800 grit SiC paper and TEM foils were prepared by twin-jet electrochemical polishing machine with the electrolyte solution consisting of 10 vol% perchloric acid and 90 vol% methanol at −30 °C. Subsequently, TEM analyses were performed on a FEI Tecnai G2 F30 S-TWIN operating at an acceleration voltage of 200 kV.

## CRediT Author Statement

**Mohsen Saboktakin Rizi:** Formal analysis, Investigation, Data curation, Writing – original draft; **Hossein Minouei:** Investigation, Resources, Validation; **Byung Ju Lee:** Investigation, Resources, Validation; **Hesam Pouraliakbar:** Investigation, Resources, Validation; **Mohammad Reza Toroghinejad:** Conceptualization, Methodology, Writing – review & editing; **Sun Ig Hong:** Conceptualization, Methodology, Writing – review & editing, Supervision.

## Declaration of Competing Interest

The authors declare that they have no known competing financial interests or personal relationships which have or could be perceived to have influenced the work reported in this article.
